# English Tube Teeth

**Published:** 1885-11

**Authors:** Lawrence Vanderpant

**Affiliations:** Orange, N. J.


### ENGLISH TUBE TEETH.

BY DR. LAWRENCE VANDERPANT, ORANGE, N. J.

Since writing my article on the above subject, I have considered that it may be deficient in some minute details that will detract from the practical value I desired it should have, but as the use of such teeth is so well known in Europe I did not at the time deem such particularity essential.

The initial process in mounting these teeth is much the same as with the American, previous to backing. They are roughly fitted, usually in pairs. The second step is the most important, "pickling," or securing the proper position of the pin, which is thus effected. The Platina tube of the tooth is cleaned by an appropriate tool called a "Tube File," and counter-sunk at its base. (A convenient instrument is made for this purpose by grinding a four-surfaced point on the end of an old rat-tail file.) The tooth is secured to the plate by a cement composed of two parts fiddler's resin and one part beeswax. The position of the pin is given by pointing the wire to be used, and placing thereon the smallest quantity of color (vermilion and oil.) By passing the wire through the tube of the tooth on to the plate, the exact center of the tube is given, and the proper position of the pin. After removing the tooth and cement, make an indentation with an improvised point in the colored spot, and drill a hole slightly smaller than the wire to be used; cut out from the inside of the plate the debris from the drill or broach (a useful gouge for this purpose can be home-made out of a half-round steel wire, three-eighths of an inch wide, ground to a rather bluff cutting edge), cut off an ample length of wire for the pin, file the end to be soldered to the plate square, and insert it firmly in the proper position in the hole already made, so that it will not require subsequent bending. The object of forming a square point is that the solder may have a substantial attachment.

Pliers are sold especially for this work, but by filing or grinding a longitudinal groove in an ordinary pair, all that is necessary is obtained. I would here remark that for this, or for crown, or clasp fitting work, I always teach my pupils to use pliers rather too large, than the reverse. Much time is lost and vexation caused by the wriggling and slipping of improper tools.

The pin or pins must now be soldered, for which the smallest possible quantity of solder and borax paint is all that is needed. In firing, pay special attention to the plate, and draw the solder from underneath as far as possible. Avoid the pins, otherwise they will "sweat," and endless trouble ensue. An inefficient solderer might paint two-thirds of the pin with "Whiting Paint," which will prevent their melting or "sweating." In like manner all the teeth are mounted and the excess of pin cut out from the inside of the plate.

After all the teeth have been perfectly fitted, which may be done most accurately by painting the plate adjacent to the pin with the oil and vermilion, place the tooth thereon, which, on removal, will have the exact mark of color of the portion requiring grinding.

The plate polished, the teeth boiled in soda and water to remove grease, etc., the tubes cleaned by passing a string through them, and the whole of the work perfectly dry, the teeth are fastened to the plate and pins by means of sulphur. Some care is necessary in effecting this, which is done by heating the work carefully over an alcohol or Bunsen burner, and placing, with a pair of foil carriers or jeweler's tweezers, a piece of broken sulphur about twice as large as a pin-head, which will flow through the tube and hermetically seal the tooth and plate. The sulphur may also be applied by making a piece of iron or other wire hot, and taking up a lump the proper size thereon. It is obvious that some care and dexterity is requisite in this, the final act, for a little injudicious application of heat will ignite the cement, and the whole of the work will be badly disfigured, and perhaps some of the teeth cracked. Ordinary care and foresight will, however, prevent such disasters. The plate should be grasped with the pliers in the left hand, and heated with a small, well-regulated flame, from the centre of the plate, about where the air-chamber usually is, and if not a particle of sulphur is allowed to drop on to the plate, a satisfactory result will be obtained. The final finish is to be effected by grinding out with an extra fine small stone the excess of the pin, when a little polishing with the fine brush wheel will remove the tarnish from the plate. Subsequent removal of the teeth, if necessary, is effected by heat in the above-described manner. The only difficulty that I apprehend a novice would encounter would be in soldering the pins, but by attention to instructions herein, and a little practice, this will be easily surmounted.

In the application of these teeth to crown or pivot work, facility and greater accuracy is obtained by cutting a screw upon the end of the pin to be inserted in the root. Two or three turns will secure it firmly in position, when it can be bent to fit the requirements of the tube-tooth, and amalgam easily be plugged around it.

